# GoodFibes: An R Package for The Detection of Muscle Fibers from diceCT Scans

**DOI:** 10.1093/iob/obad030

**Published:** 2023-08-17

**Authors:** J H Arbour

**Affiliations:** Department of Biology, Middle Tennessee State University, Murfreesboro, 37132, TN, USA

## Abstract

Contrast enhanced computed-tomography imaging like diffusible iodine-based contrast-enhanced computed tomography (diceCT) can provide detailed information on muscle architecture important to comparative analyses of functional morphology, using non-destructive approaches. However, manual segmentation of muscle fascicles/fibers is time-consuming, and automated approaches are at times inaccessible and unaffordable. Here, we introduce GoodFibes, an R package for reconstructing muscle architecture in 3D from diceCT image stacks. GoodFibes uses textural analysis of image grayscale values to track straight or curved fiber paths through a muscle image stack. Accessory functions provide quality checking, fiber merging, and 3D visualization and export capabilities. We demonstrate the utility and effectiveness of GoodFibes using two datasets, from an ant and bat diceCT scans. In both cases, GoodFibes provides reliable measurements of mean fiber length compared to traditional approaches, and is as effective as currently available software packages. This open-source, free to use software package will help to improve access to tools in the analysis of muscle fiber anatomy using diceCT scans. The flexible and transparent R-language environment allows other users to build on the functions described here and permits direct statistical analysis of the resulting fiber metrics. We hope that this will increase the number of comparative and evolutionary studies incorporating these rich and functionally important datasets.

## Introduction

**Fig. 1:**
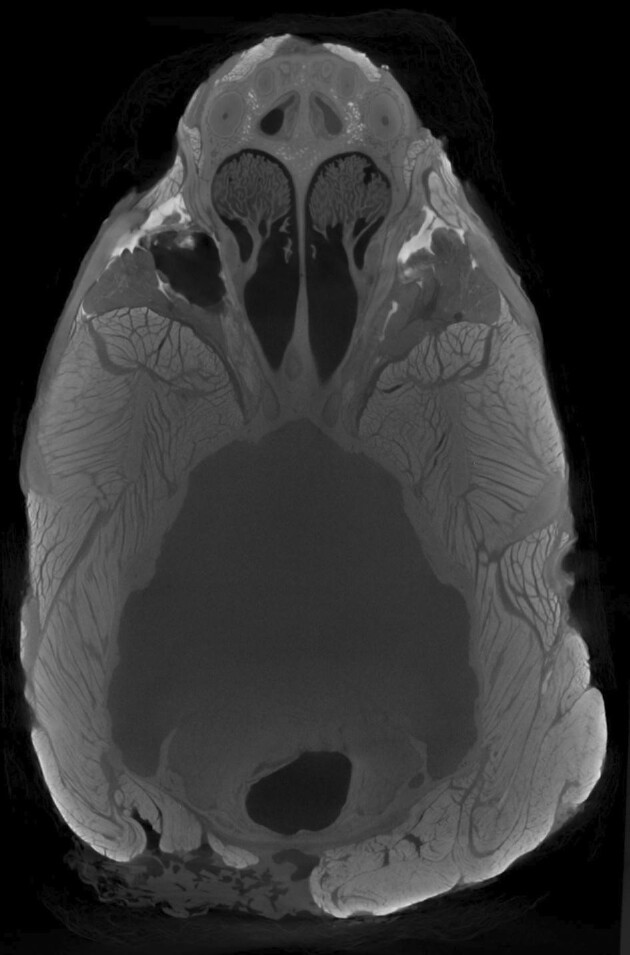
A diceCT scan of a North American river otter (*Lontra canadensis*, MEV0216) skinned head stained for 106 days in Lugol's iodine (1%). Scanned at 160 kV and 670 µA in a North Star Imaging System. Axial view showing visible muscle fascicles. Also, see B in Fig. 3.

While traditional approaches for anatomical and biomechanical studies relied primarily on destructive techniques like dissection, increases in accessibility of computed-tomography (CT) scanning resources have ushered in a renaissance of 3D biological imaging for comparative and biomechanical studies (e.g., Gignac and Santana 2016; Gignac et al. 2016; Muñoz and Price 2019). CT scanning has long been used to visualize hard tissue structures like bones, however, more recently diceCT (diffusible iodine-based contrast-enhanced computed tomography), has been applied to the fine scale resolution of soft tissue structures like muscles and brains/nerves (Fig. 1; Kupczik et al. 2015; Gignac et al. 2016; Gignac and Kley 2018; Santana 2018; Dickinson et al. 2019; Sullivan et al. 2019; Camilieri-Asch et al. 2020; Holliday et al. 2022).

In particular, diceCT may be particularly useful in the analysis of muscle tissue, as muscle fascicles can often be resolved (Fig. 1), permitting the collection of muscle architecture data, such as fiber length and pennation angle (Dickinson et al. 2018; Sullivan et al. 2019; Katzke et al. 2022). Such variables are critical in estimating muscle strength and function, for example, in the calculation of physiological cross sectional area (Herrel et al. 2008). However, the quantitative analysis of muscle architecture at the fiber/fascicle scale has been limited, especially compared to the abundant comparative analysis of skeletal shape from CT scanning projects (Buser et al. 2018; Arbour et al. 2019; Paluh et al. 2020; among many other examples; Evans et al. 2021). Manual segmentation of muscle fibers is tedious, time consuming, requires an expert knowledge of muscle anatomy, and is not feasible for all datasets (Dickinson et al. 2019). While automated approaches exist (Kupczik et al. 2015; Dickinson et al. 2018; Sullivan et al. 2019; Püffel et al. 2021), their lack of widespread adaptation is partly due to the accessibility and cost associated with software options available.

Katzke et al. (2022) provided a thorough review and comparison of the effectiveness of all major approaches for the 3D reconstruction of muscle fibers currently available. But to briefly summarize the major available methods: The Xfiber module available for distribution with **Avizo/Amira** uses cylindrical template-based fiber tracking approach, and has been effectively applied to vertebrate datasets like crocodiles and birds (Sullivan et al. 2019; Holliday et al. 2022). This proprietary software package is costly, and while generally effective is sensitive to the input parameters and assumes that fibers have consistent diameter (Sullivan et al. 2019). The **ImageXd** software package described by Kupczik et al. (2015), is a standalone command line application that applies textural analysis (i.e., variation in grayscale values) and reconstructs muscle fibers from streamlines of tensors. Puffel et al. (2021) produced a stepwise approach in Python and Blender, which was developed to reconstruct straight-path fibers. This approach projects a trajectory down a muscle fiber based on the path of lowest grayscale variation, and was specifically developed for processing an ant muscle dataset; the intersection of the muscle with the apodeme of the exoskeleton must be known. Additionally, this approach cannot currently accommodate curved fibers commonly found in vertebrate muscle. Regardless of the different approaches used here, these methods produce similar results in terms of fiber length and pennation angle to each other, though vary in effectiveness across datasets (Kupczik et al. 2015; Dickinson et al. 2018, 2019; Sullivan et al. 2019; Katzke et al. 2022).

Here, we introduce an R-language (v4.3.0, 2023) based software package (GoodFibes; Arbour 2023) for the reconstruction of muscle fibers from diceCT datasets that incorporates aspects of the previously described methods, while making these approaches available in a widely used, open-source language. As input GoodFibes requires a sequential stack of grayscale images in PNG format, which can be exported from many CT reconstruction software packages or converted from other formats using ImageJ. The approach described here uses variation in grayscale values to track muscle fibers, under the assumption that variation in voxel radiopacity should be minimized along the fascicle/fiber track, similar to ImageXd and the approach described in Puffel et al. (2021). GoodFibes uses an iterative approach like Puffel et al. (2021), however, is able to change trajectories with each step.

The main function of this package (good.fibes) is constructed such that only a small stack of images (= radius + 1, see arguments described below) are held in working memory in R at any given time. This drastically reduces the burden on memory usage, which can be quite high for large, micro-CT datasets consisting of 1000+ images and is often a major limiting factor in the analysis of such datasets. However, it does trade-off in decreasing the computational efficiency of the tracking algorithm, as images must be read in and removed repeatedly. Given the computational times observed by the author for several microCT datasets (and see example 1), this is a reasonable trade-off for a typically sized dataset. Here, we show that this approach performs similarly to previously mentioned software (Amira, ImageXd, Puffel et al. 2021) in the estimation of fiber architecture and the quantification of fiber length in particular.

## Methods

**Fig. 2:**
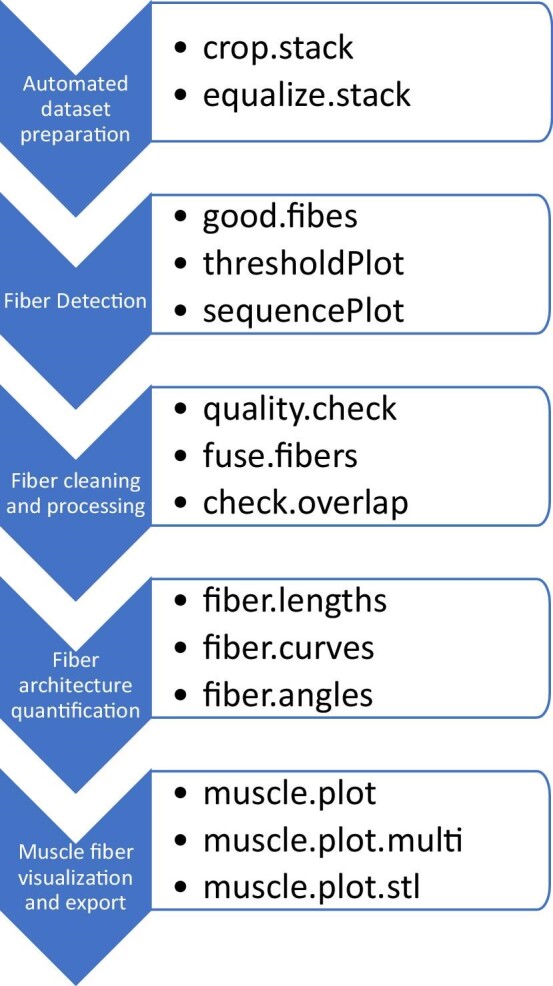
Workflow for the use of the GoodFibes package and major functions.

The application of the major functions in this new package is illustrated in Fig. 2. Automated cropping (crop.stack) and equalization (equalize.stack) can be completed on the muscle stack, however, more advanced image adjustments can be made using the free software ImageJ. In particular, the tools “Process > Math > Gamma” and “Process > Filters > Unsharp Mask” in ImageJ/Fiji are effective at reducing noise and improving contrast between muscle fascicles (Jaimi Gray, pers. comm.). The automated cropping function (crop.stack) will ensure a sufficient buffer region of black voxels is present around the muscle to ensure the tracking algorithm will be able to evaluate all possible paths (see below). Subsequent functions are provided to help choose initial parameters (thresholdPlot), tracking muscle fibers (good.fibes), and visualizing the sequence in a tracked fiber (sequencePlot) (and see details below). Several functions are provided for cleaning, quality-checking and combining detected muscle fibers, and for quantifying metrics like length and tortuosity (curvature). Lastly, reconstructed muscle fibers can be visualized and exported as stereolithography; triangular surface mesh (STL) meshes.

The main function of this package, “good.fibes,” uses as input a sequential image stack (.png) containing only the grayscale values for the muscle of interest. The image stack must be isometric (the distance between images is the same as the pixel dimensions) and must occur in sequence in the folder (and when accessing image files names with list.files—see example code). Only one muscle can be analyzed at any given time, and multiple muscles must have their own individual image stack (and see the bat dataset in empirical examples). Images are converted to R compatible objects using the package imager (Barthelme 2021). The muscle “region of interest” (region of interest (ROI)—i.e., the pixels representing the muscle in each image of the original scan) can be manually segmented through any available segmentation software that will allow the export of the substack (the complete grayscale data for just the muscle) or a binary ROI (an image stack with white representing the region of interest/muscle and black for all other pixels), such as Dragonfly ORS (“Dragonfly ORS” 2022), Mimics (Materalise), Amira/Avizo (Thermo Fisher Scientific), or 3D Slicer (Kikinis et al. 2014) (among others). For binary ROIs, the grayscale values can be isolated by importing both image stacks (original grayscale data and the binary ROI) into ImageJ and using the “Image Calculator” tool (under “Process”). In our experience processing stacks exported from Dragonfly, we first subtracted the original image stack **from** the binary ROI/muscle mask image stack, and then subtracted the resulting stack from the binary ROI again. This produced a stack with grayscale values between 0 (black) and 1 (white), with muscle being represented by gray values (between 0 and 1).

The fiber tracking function “good.fibes” proceeds by first (1) determining a set of well-spaced seed points within an image plane, each of which will (potentially) produce a single muscle fiber (but see below), (2) completing a stepwise forward and backward walk from that seed point, and (3) smoothing the fiber path using splines. Subsequently, functions may be used to assess the quality of detected potential muscle fibers/fascicles, combining partial fiber paths or removing redundant ones, visualizing muscle fibers in 3D, and calculation of fiber length, orientation, and curvature. The order of use of the various functions available is shown in the flowchart in Fig. 2.

### Selection of seed points

The main function is designed to automatically select a set of well-spaced seed points across a plane of interest (image plane and number of seed points are user selected). We applied a hierarchical cluster analysis (using “UPGMA”) of a Euclidean distance matrix of all greyscale points above a threshold value, using the R function “hclust” (package “stats”; R-Core 2023). This “threshold argument”—(values from 0 to 1, black to white, corresponding to the brightness of a voxel), is intended to isolate “bright” voxels with a high likelihood of representing muscle fibers. The initial value for threshold (and related parameter cutoff, see below) can be chosen after viewing different options with “thresholdPlot” (and see supplementary code). This step is computationally intensive, and increasing the threshold value can help to reduce runtime especially for very large image files. However, very high values for threshold may eliminate major portions of the muscle, especially if there is a slight gradient in grayscale values from staining, and thus fibers detected would not be representative of the whole muscle.

The dendrogram produced was split into a number of selected clusters equal to the number of required seed points (argument “seed”) using the R function “cutree.” A single starting point was randomly selected from each cluster. We found that this procedure produces a series of starting points for the tracking algorithm, which will be well distributed throughout the muscle, but represent random starting positions. The argument “start.seed” can be used to set the same seed point for repeatable results. The number of muscle fibers detected will be less than or equal to the value for seed, as some starting points may represent noise with no possible pathway forward or backward. In these cases that seed point is dropped and no muscle fiber is produced from that location.

### Tracking algorithm

From the selected seed point, the tracking algorithm moves forward and backward through the image stack. To reduce noise and remove values that do not represent muscle tissue, all grayscale values (typically ranging between 0 and 1, for black to white respectively) below a certain threshold value (argument “cutoff,” which can be identical to argument “threshold” or lower/less restrictive) are set to zero (“black”). Setting “threshold” higher than “cutoff” (i.e., threshold considers fewer voxels as non-black) can minimize the time it takes for seed points to be selected, as this step is computationally intensive, and help to localize seed points in bright areas central to muscle fascicles and unlikely to represent interstitial space. However, “threshold” and “cutoff” may be supplied as the same value, especially in scans with good resolution and little noise. In the author's experience these values should be set conservatively/harshly (higher) to help the algorithm follow within one muscle fiber. Very short fibers or poor quality tracks can be filtered out later using “quality.check” or combined with “fuse.fibers,” but tracks that wander between muscle fascicles are more difficult to correct.

From the selected seed point, a hemisphere/spherical cap of points is projected through the next sequence of images in the image stack. The dimensions of the hemisphere are determined by the value radius, which can currently be set up to 11 (though larger values create more possible paths). For most datasets a value between 5 and 9 will offer a reasonable number of paths that can be evaluated efficiently, with very curved fibers benefited by smaller values of radius. An option (“backstep”) allows paths within the same image plane as the seed point, or “behind” the seed point in the image stack to be considered in the spherical cap of possible paths. This permits particularly curved fibers that pass back through the image stack (e.g., see worked example 2) to be reconstructed, but may increase noise by allowing lateral movement with a muscle fascicle.

To evaluate the possible paths from the seed point, the standard deviation (SD) in gray values is calculated from all possible unique straight-line paths. Muscle fibers tend to be arranged without abrupt changes in trajectory (generally are straight or curving, as opposed to sharp bends). To account for this, we calculated the deviation of the path from the proceeding trajectory as the Euclidean distance between the end points, scaled the values to lie between 0 and 1, and added this value to 1 (resulting values range from 1 to 2). The selected path was chosen as the path that minimized the following: diagnostic value = scaled grayscale SD * trajectory^scalar^. Paths with low grayscale variation and that follow a consistent trajectory through the image stack will produce the lowest diagnostic values and be selected. The scalar allows users to tailor the impact of trajectory changes (for example, a scalar of zero removes the trajectory penalization). Once the best path is selected (that minimized the above diagnostic value), the algorithm resets and a new hemisphere of paths is selected.

The tracking algorithm continues this stepwise process until it reaches one of several stop conditions. These conditions are intended to prevent the tracker from passing out of a muscle fascicle and to terminate when reaching the end of the muscle fiber. The conditions under which the backward/forward walk from the seed point terminates include: (1) The only available remaining paths would either terminate or cross a black voxel. This prevents the tracker from passing out of the muscle fascicle. To accommodate noisy datasets, the tracker may be permitted to cross a small number of black voxels. (2) The number of black voxels in the possible paths exceeds a specified number (e.g., 95%). This is meant to isolate regions of noise toward the end of a muscle fascicle, where adjoining connective tissue may obscure the end of a fiber. (3) The remaining paths would terminate within a specified distance of the external “boundary” of the muscle (argument “bound.buffer,” suggested range of 2–5 voxels depending on image size). This prevents fibers from continuing to track along any connective tissue surrounding the muscle in noisy image stacks and to help with compressed boundaries, which can be observed in tightly packed specimens. The bound.buffer argument is particularly important in vertebrate muscle datasets like the otter, bat and darter shown, and less important in the well-defined fibers of the ant dataset.

Once the tracker has reached a termination condition, it returns to the seed point and proceeds in the opposite direction through the image stack, using the exact same parameters described above. The forward and backward walks through the image stack are then merged into one continuous path, and the function moves to a new seed point.

### Output and smoothing of muscle fibers

The function good.fibes returns a list object (for each seed point) that contains ${\$}$fiber.points (the raw step-wise coordinates the tracking algorithm returned). Reconstructed fibers from “good.fibes” initially comprise straight line steps. The function “smoothed.fibers” (and several data processing function, like fiber.lengths) use natural cubic splines as implemented by function “nsp” (package “splines2”; Wang and Yan 2021), which are piecewise polynomial functions defined by a number of breakpoints or “knots,” to smooth these stepwise tracks into realistically curved muscle fibers. Such splines can be defined using “knots” or “degrees of freedom” (knots = df–1), where “nsp” selects the knots at a suitable location for a shift in the polynomial function (i.e., a bend). Values over df > 1 produce increasingly curved fiber paths, where df = 2 can produce an arched or “U” shape, df = 3 can produce “U” or “S” shaped paths, and so on. In the case of the straight fibers of the ant dataset below a df = 1 was used, and for the vertebrate datasets df = 2 was used as these had simple arching paths between origin and insertion locations. It is likely that df values between 2 and 4 would be sufficiently curved/“bendy” to accommodate most vertebrate muscle fibers.

### Processing and checking of reconstructed muscle fibers

We assessed the quality of fibers produced by “good.fibes” following the metrics outlined by Puffel et al. (2021)—the ratio between the variation in grayscale values along a fiber track to the length of the reconstructed fiber. Those fibers with high variability compared to fiber length (and determined to be outliers) were removed from further analysis by the function “quality.check.” Optionally, fibers below a set length can also be excluded (similar to the ImageXd and Amira approaches), based on, for example, overall dimensions of the original muscle volume.

Following removal of poor-quality fiber paths, two functions permit the merging of separate fiber tracks. This is meant to help combine paths from a single otherwise incomplete fiber that did not complete the full walk along its path due to noise or other irregularities in the dataset. The function “fuse.fibers” will combine two fibers into a single fiber path, if the new path is improved compared to the two separate paths (in length and mean residuals) and the function “check.overlap” is used to remove redundant fibers, produced from separate seed points within the same fascicle or fiber but resulting in slightly different fiber tracks. Both of these evaluate two fibers if they fall within a minimum distance in voxels (min.vox) of each other. This should be selected based on the width of the fascicles and the distance between fascicles, but we generally used ∼½ of an average fascicle width in cross section.

### Quantification and visualization

Package GoodFibes currently carries out the calculation of fiber length, orientation and curvature (tortuosity), based on spline-smooth fiber paths, via the functions “fiber.lengths,” “fiber.angle,” (in degrees) and “fiber.curve,” respectively. Fiber length can be output in units of voxel length or in the actual scale of the original scan data by providing the scan resolution (res). Orientation is currently in comparison to a specific axis (similar to the Katzke et al. 2022 visualization), but future updates to the package will permit the comparisons of fibers to a tendon.

**Fig. 3:**
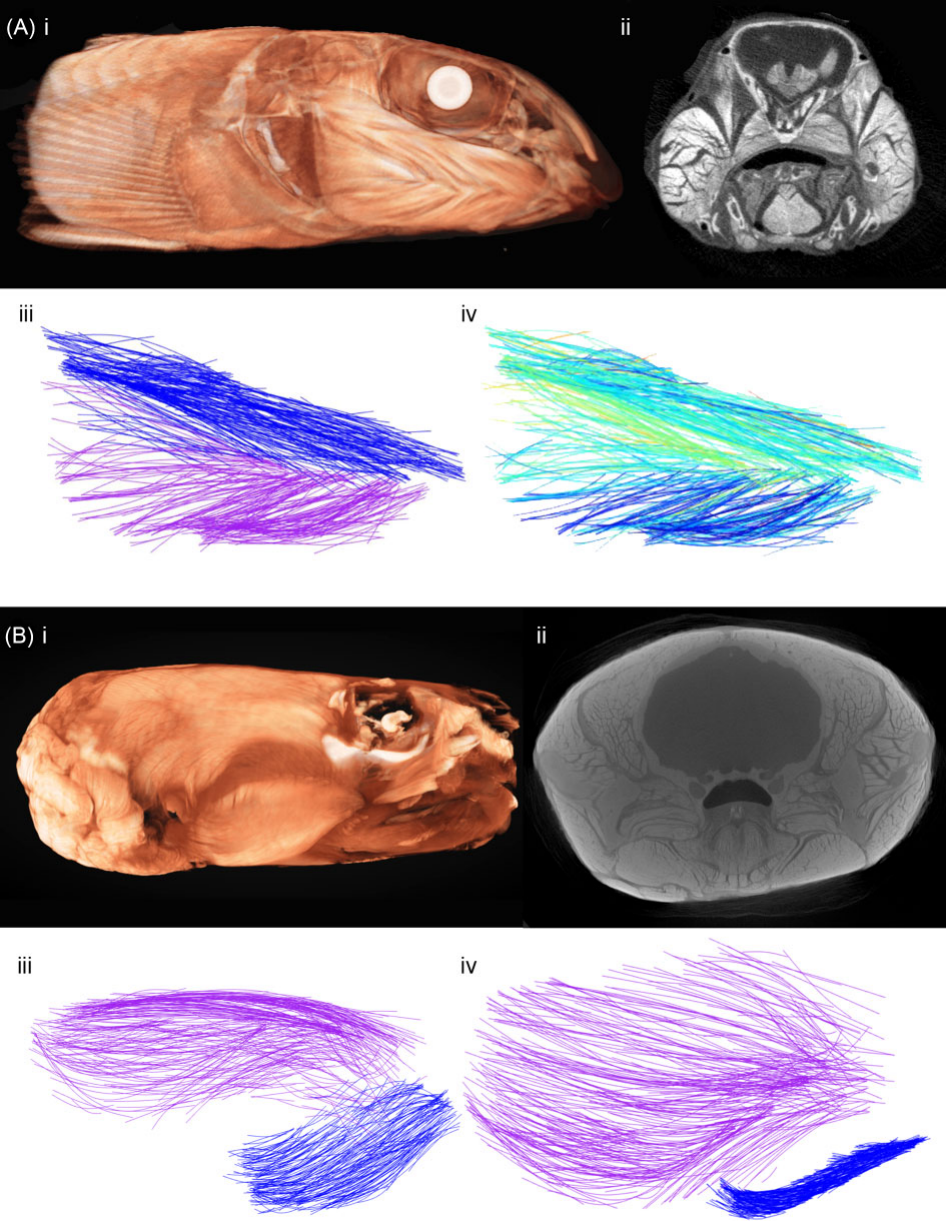
Sample datasets processed using the GoodFibes package, showing a range of size, taxa, and visualizations. (A) Greenside darter (*Etheostoma blennioides*, MTSUZ-9) diceCT in coronal view (17.2 µm, stained with Lugol's iodine 1% for 3 days, scanned whole in a Scanco µCT50 system at 55 kV and 200 µA, at the Vanderbilt Center for Small Animal Imaging). (i) 3D reconstruction in Dragonfly ORS and (ii) single sample image from stack. The two major divisions of the adductor mandibular (AM1 and AM2) were segmented and muscle fascicles automatically detected. Visualized with “muscle.plot.stl,” (iii) showing the divisions labelled by colors and (iv) colored by orientation compared to the central axis. (B) North American river otter (*Lontra canadensis MEV0216*) head diceCT (70.8 µm, stained for 106 days, and scanned at 160 kV and 670 µA on a North Star Imaging System at the University of Washington). Specimen was skinned prior to staining. (i) 3D reconstruction of scan and (ii) single image from stack. Muscle fibers from the temporalis (purple) and superficial masseter (blue) in (iii) lateral and (iv) dorsal views.

**Fig. 4:**
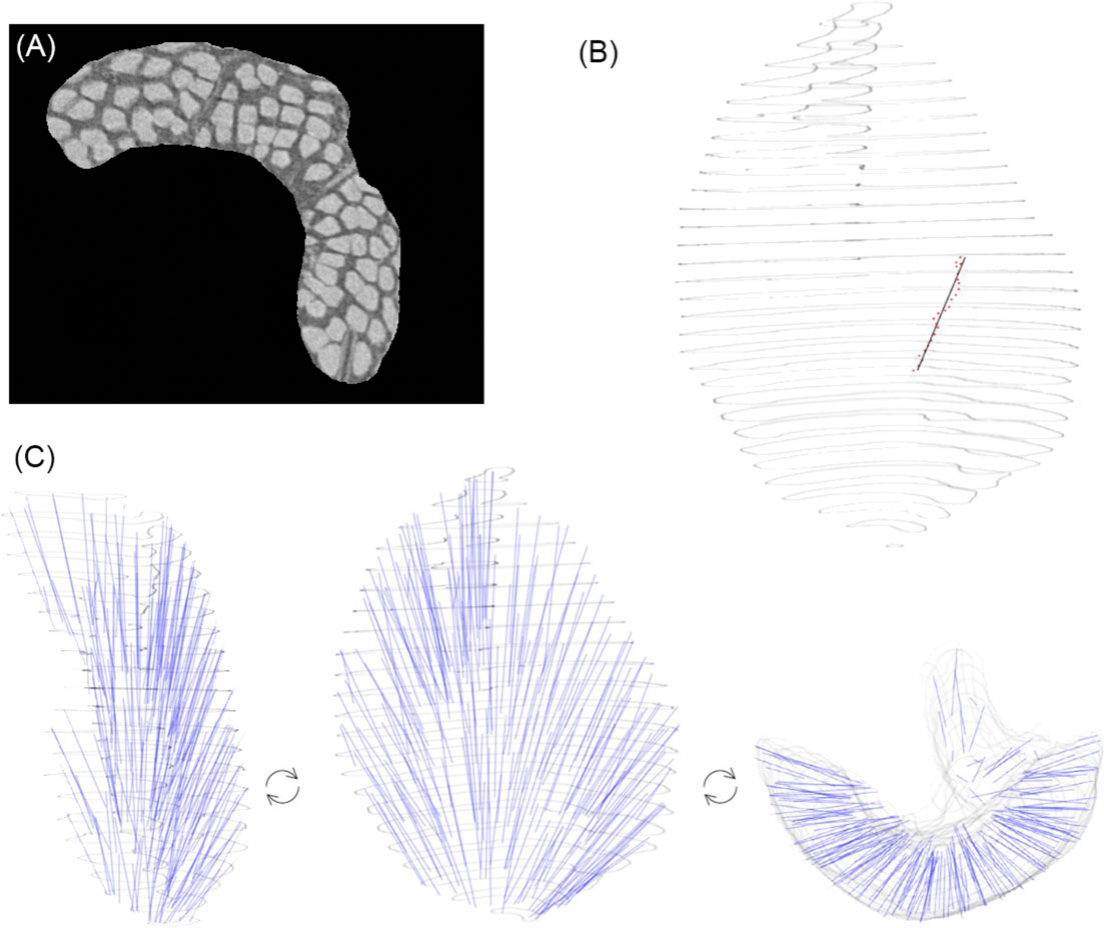
Mandibular muscle in an ant (*Monomorium pharonis*). See Katzke et al. (2022) for scanning parameters and other details. (A) Image from diceCT image stack, with muscle ROI segmented. Muscle fibers were oriented to pass through the image stack, rather than parallel to the image plane. (B) A single fiber track (points) and the smoothed path through the muscle, generated from a single automatically determined seed point. (C) A total of 198 muscle fibers detected and processed in GoodFibes, shown from three angles. Dataset from Katzke et al. (2022), and detected fibers available as “ant.final” data object.

Visualization of 3D muscle fibers draws largely from the capabilities of the R package “rgl” (Adler et al. 2003), and see Fig. 3. Correspondence between the original muscle fiber “track” and the smoothed fiber can be visualized using “muscle.plot” (Fig. 4). Processed fibers can be illustrated along with a boundary showing the muscle shape using “muscle.plot.multi” (Fig. 3). Finally, “muscle.plot.stl” can be used to export the 3D muscle fibers as a triangular surface mesh STL (.stl) file, with the option to first check the visual properties (save.plot = FALSE). If the resolution of the scan is provided, the muscle fibers will be scaled to actual size in the 3D mesh file and these can be opened in any 3D mesh editing software like “Geomagic,” “Meshmixer,” or “Blender” (among others). The visualization in Fig. 6 was produced by exporting the bat muscles as individual STL files and importing them into Geomagic. The skull in this image was produced from a separate, non-stained scan and manually aligned with the generated muscle groups (based on dissection photos and anatomical landmarks) in Geomagic, as is already commonly done with volume reconstructions of muscles in other diceCT works (Santana 2018).

If multiple muscles are processed the results can be combined both within and outside of the R language environment. If all muscles are generated from stacks representing the same “space” (i.e., in the same view/plane, without cropping, and all images in the sequence are in each stack), then they will be automatically aligned in 3D coordinate space (with each other) and can be plotted together in R (as well as after export to programs like Geomagic and MeshLab). Using the concatenate function (“c”) the lists containing the two muscle fibers can be combined into one object for plotting (e.g., with “muscle.plot.stl”), while a vector of colors can be provided based on muscle group or other properties like length (Fig. 3, and see Supp. Mat. for example code). If the data are cropped prior to muscle fiber detection (either by image or fully black images were first removed), then values can be added to the fiber coordinates to realign the muscles (examples given in supplementary materials). The function “crop.stack” will output the necessary values for the x and y coordinates (xlim, ylim) and the number of deleted images at the beginning of the stack can be used to provide the z value if needed, and can be rerun on the original data again (without saving images) to reacquire these values if not recorded on the first use of the function. Once the impact of cropping has been corrected, the different muscles can be combined with “c” as described above and exported as anatomically aligned STL files.

### Empirical examples

Katzke et al. (2022) use a diceCT dataset of an ant (*Monomorium pharonis*) mandibular muscle (resolution 0.67 µm) to demonstrate the effectiveness of the three previously listed applications in the estimation of fiber length and angle. This scan is high resolution, shows low noise, and the muscle fibers can be individually visualized, permitting a comparison to manually segmented values. The authors of that study kindly shared their dataset allowing a direct comparison. The muscle ROI was segmented using Dragonfly ORS software and the ROI was exported as a binary image stack. I used the image calculator functions in ImageJ to create an image stack of exclusively grayscale data for the muscle ROI, saved as .png format files. Using good.fibes, 250 seed points were applied across 5 image planes (50 each) to reconstruct muscle fibers across the image stack (threshold = 0.7, cutoff = 0.65, backstep = 0, allowed.black = 0, scaler = 1). We applied quality.check to remove fibers of low resolution, and check.overlap and fuse.fibers to combine fibers that were (1) redundant from seed points on different planes, or (2) terminated early but continued from a different starting point. We used a distance of 8 voxels in fuse.fibers and check.overlap (min.vox), as this was about half the width of a typical fiber in cross-section, so that fibers from different bundles would not be grouped together. No fibers were manually removed from the dataset. Runtime for 250 seed points was 92 min on an HP laptop with an Intel(R) i5-10300H CPU (2.50 GHz) with RAM utilization of <1 GB. Example code is given in the help files associated with the various functions in the package (see supplementary files). Muscle fiber lengths were compared with the previous studies estimates from manual digital dissection and ImageXd and Amira.

Image stacks were produced for the superficial, zygomatic, deep and medial temporalis, masseter, zygomaticomandibularis (ZM), and medial pterygoid from a diceCT scan of an Egyptian Fruit Bat (*Rousettus aegyptiacus*) stained for 28 days in 1% Lugol's Iodine, and scanned at a resolution = 10.05 µm and downsampled to 20.1 µm for processing in Mimics). Between 200 and 400 seed points across 4–6 images were used per muscle, with threshold and cutoff values visually selected using “thresholdPlot” (other arguments used across muscles: radius = 7, allowed.black = 1). Data cleaning and processing (with smoothing df = 2) follows that of the ant example, and a small number of fibers showing unusual curvature or orientation were removed (<10 per muscle), following Sullivan et al. (2019). The tracking algorithm performed inconsistently when muscle fibers lay parallel to the image plane, so the deep temporalis and ZM were reprojected in Dragonfly ORS to an axial view (using the data inverter plugin > axis transformation prior to exporting the binary image stack), while all other muscles were analyzed in coronal view. For the superficial temporalis and ZM, some fibers curve or are reoriented back into the image plane, and backstep = 1 was included. Mean fiber lengths were compared with those taken from traditional dissection (*n* = 15 per muscle) from previous (unpublished) work on bat bite biomechanics. For dissection and fiber separation protocol see Hartstone-Rose et al. (2018).

## Results

**Fig. 5:**
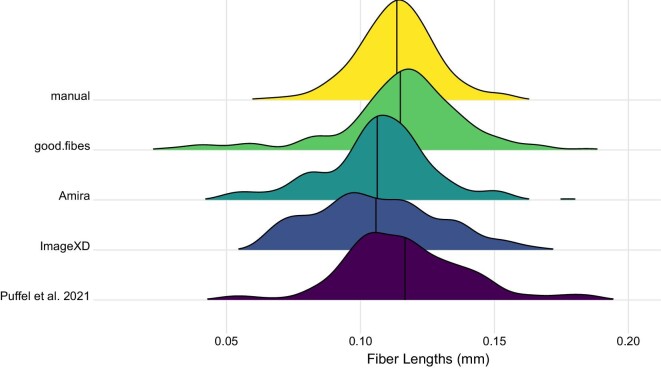
Comparison of GoodFibes to currently available approaches (see introduction for descriptions) on the ant mandibular muscle dataset. Data for manual, Amira, ImageXd, and Puffel et al. 2021 taken from Katzke et al. (2022). Vertical line indicates mean value per method.

Both empirical datasets show reasonable effectiveness in estimating fiber lengths compared with previous software applications and traditional approaches. After application of “quality.check,” “fuse.fibers,” and “check.overlap,” a total of 198 smoothed muscle fiber paths (df = 1) were produced for the ant muscle. Reconstruction of muscle fibers in 3D appears visually similar to that produced in the prior study (Fig. 4). The mean fiber length calculated using “fiber.lengths” was 0.115 mm (sd = 0.025 mm). This was well matched to the mean length from manual segmentation reported by Katzke et al. (2022), (mean = 0.114 mm, sd = 0.014 mm) and fell well within the estimates produced by other methods (0.106–0.117 mm, Fig. 5). Minimum fiber length was somewhat lower in our approach, although several of the other methods use a minimum fiber length cutoff as part of their reconstruction approach (e.g., Amira).

**Fig. 6:**
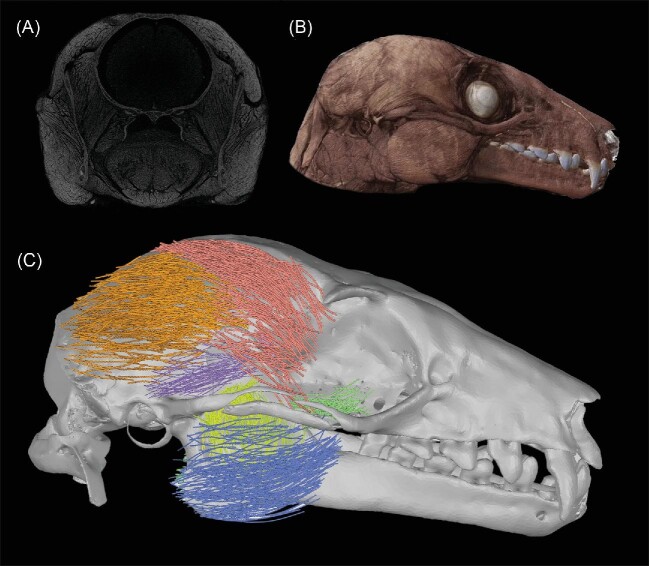
Muscle fibers in *Rousettus aegyptiacus*, from the research collection of Sharlene Santana, University of Washington. (A) Original sample diceCT image, scanned on a Skyscan 1172 at 60 kV, shown in coronal view. (B) 3D Visualization of diceCT scan data. (C) Muscle fibers automatically detected by GoodFibes (red = s. temporalis, orange = m. temporalis, purple = z. temporalis, pink = d. temporalis, blue = masseter, yellow = ZM, green = pterygoid. Fibers were exported from R using “muscle.plot.stl” as STL files and imaged with the skull in Geomagic, see methods (“visualization”) for details.

In the case of the cranial adductors in the Egyptian fruit bat, muscle fiber reconstructions were visually consistent with the diceCT reconstruction (Fig. 6) and as observed during the muscle dissections. Mean fiber lengths were strongly correlated with those produced by manual dissection (r = 0.86) and deviations were typically small (<10% of average fiber length; Table 1). The superficial temporalis showed the greatest departure, though this was still within the performance range of previous approaches (e.g., Sullivan et al. 2019). This may result from the orientation of the superficial temporalis, which in the image stack makes a sharp “downwards” turn as it approaches its attachment site.

**Table 1:** Mean fiber lengths produced from manual dissection (*n* = 15) and reconstruction. Percent difference calculated from manual dissection estimates

	Manual	Digital reconstruction	Percent
	dissection	via GoodFibes	difference
Masseter	6.905	7.335	5.86%
Medial pterygoid	3.538	3.82	7.38%
Deep temporalis	6.223	5.586	−11.40%
Medial temporalis	6.427	7.018	8.42%
Superficial temporalis	7.877	6.481	−21.54%
Zygomatic temporalis	5.722	5.905	3.10%
Zygomaticomandibularis	5.34	5.209	−2.51%

## Discussion

Overall, GoodFibes presents a novel combination of features in automated muscle fiber detection, and produces reasonable reconstructions of muscle fascicles/fibers in two worked examples. The range of deviations from dissected muscle fiber lengths our approach (2.5–21.5%) was within the range observed for ImageXD and Amira from other examples in vertebrate muscle. Sullivan et al. (2019), found a deviation of 23% in mean fiber length using Amira in a starling pectoral muscle, while ImageXd produced differences between 8 and 42% in primate jaw muscles (Kupczik et al. 2015; Dickinson et al. 2018). Manual segmentation of fascicles from diceCT scans produced comparable differences to GoodFibes (2.3–18.0%; Dickinson et al. 2019). We also note that our estimate of mean length from manual dissection in the Egyptian Fruit Bat is based on only 15 fibers per muscle. While this is typical practice for many biomechanics studies in bats and other mammals, this small sample is also likely to vary from the true parameter mean of the muscle.

Like any approach using diceCT scan data to reconstruct muscle fibers, the effectiveness of this approach will be limited by the quality of the scan data, including: having an appropriately high-resolution scan with good contrast between muscle tissue and connective tissue; having no major artifacts in the scan (e.g., ring artifacts); having even perfusion of stain throughout a segmented muscle. Muscles with uneven staining (i.e., brighter outer regions of the muscle tissue on scans) may result in the tracking algorithm choosing paths that move between fascicles in brighter regions and having more paths end abruptly in less stained regions due to thresholding. Similar to the Amira fiber tracking module, GoodFibes requires estimation of a set of grayscale thresholds, and other input parameters like the radius of each step in the tracking algorithm, etc. In both cases there is no currently published approach for determining a reasonable set of starting values, and thus for both approaches the selection of these values is through subjective user assessment.

As noted for the deep temporalis and ZM muscle from the bat dataset, the tracking algorithm performed more inconsistently when muscle fibers were oriented with the plane of the images (results not shown). However, exporting the image stack for these muscles in a different plane was sufficient to produce reasonable estimates of fiber length. Muscles with sections that vary substantially in orientation, such that some muscle fibers are oriented within the image plane, while others are more orthogonal to it (e.g., superficial temporalis), might produce a biased sample of fiber lengths (this muscle produces the largest discrepancy). In such a case, segmenting the regions of the muscle into individual stacks or reorienting the image stack could mitigate these issues, but other software options (e.g., Amira which uses template-based tracking), may be more suitable in these cases.

GoodFibes provides reliable reconstruction of muscle fibers from diceCT scans in a flexible and open-source R language environment. While we have not evaluated this approach on datasets produced for other staining approaches (e.g., Phosphotungstic Acid), it is possible it could be effectively applied in these cases as well, but a separate evaluation with known (fiber length) datasets would be advisable. We hope that this package will address long-standing issues regarding accessibility and affordability in the automated detection of muscle fibers, and facilitate greater comparative and macroevolutionary analysis of muscle architecture from such non-destructive approaches.

## Supplementary Material

obad030_Supplemental_Files

## Data Accessibility

All functions required for the above analyses are distributed with the package GoodFibes available through CRAN (https://cran.r-project.org/web/packages/GoodFibes). The ant dataset is available via Dryad with the original publication Katzke et al. (2022), and the segmented and reoriented files are hosted on github.com and accessible through the help files in the GoodFibes documentation. The finalized muscle fibers for the greenside darter and otter are available through the supplementary materials. The bat diceCT image stack is available via Morphosource (media ID: 000,541,794), the STLs are available in the supplementary material and a color coded 3D model is viewable through the author's sketchfab account (https://sketchfab.com/MTSUichthyology).
